# Effects of yeast extract supplemented in diet on growth performance, digestibility, intestinal histology, and the antioxidant capacity of the juvenile turbot (*Scophthalmus maximus*)

**DOI:** 10.3389/fphys.2024.1329721

**Published:** 2024-01-24

**Authors:** Jingwu Sun, Yahui Li, Tiancong Ren, Qian Gao, Lingqi Yin, Yunzhi Liang, Haiyan Liu

**Affiliations:** ^1^ College of Future Information and Technology, Shijiazhuang University, Shijiazhuang, China; ^2^ Hebei Key Laboratory of Animal Physiology, Biochemistry and Molecular Biology, College of Life Sciences, Hebei Normal University, Shijiazhuang, China; ^3^ College of Resource and Environment Sciences, Shijiazhuang University, Shijiazhuang, China; ^4^ Hebei Collaborative Innovation Center for Eco-Environment, Shijiazhuang, China

**Keywords:** yeast extract, growth, feed utilization, digestibility, intestinal histology, antioxidant capacity

## Abstract

An 8-week feeding experiment was conducted on the juvenile turbot *(Scophthalmus maximus*) to evaluate the influence of yeast extract (YE) supplementation in the diet on growth performance, feed utilization, body composition, nutrient digestibility, intestinal histology, and antioxidant capacity. Four experimental diets were formulated with graded levels of yeast extract 0 (YE0), 1% (YE1), 3% (YE3), and 5% (YE5) and fed to turbots (initial body weight: 4.2 ± 0.1 g) with three replicates per diet and 200 fish in each replicate, respectively. The results showed that turbots fed with diets YE1 and YE3 displayed a significantly higher specific growth rate and protein efficiency rate than those fed with diets YE0 and YE5, while the feed conversion ratios in YE1 and YE3 groups were lower than those in YE0 and YE5. Fish fed with diets YE3 and YE5 showed higher body crude protein contents than those in groups YE0 and YE1. The highest apparent digestibility coefficients for dry matter and crude protein, digestive enzyme activities (trypsin, lipase, and amylase), and the height of the intestinal fold were observed in the YE3 group. YE3 treatment displayed a significantly higher superoxide dismutase (SOD) activity than the YE0 group, while the malondialdehyde (MDA) content in YE1 was significantly lower than those in YE0 and YE5. No significant difference was observed in serum physiological and biochemical parameters among all treatments. Overall, appropriate dietary supplementation of the yeast extract could improve the growth performance, digestibility, and antioxidant capacity of the juvenile turbot, and the recommended yeast extract level in the feed is 2.47%.

## 1 Introduction

Global aquaculture production has currently achieved a record high level and is expected to become more significant in the future as a source of food and nutrition for humans (FAO, 2022). However, the limited natural resources and environmental issues have become the bottleneck for the further development of aquaculture. The supply of fish meal for aquafeed is continuing to decrease, and the aquatic environment is deteriorating with the rapid rise of aquaculture (Han et al., 2018). Generally, aquatic feed accounts for 50%–60% of the total cost in aquaculture (Hardy, 2010), and its quality is closely related to the production efficiency of aquaculture and the output of waste discharged into surroundings (Hossain et al., 2023). Hence, successful sustainable aquaculture in the future heavily depends on the efficiency of the artificial feed. Yeast products are considered to be potential feed ingredients or additives that can boost fish growth performance and enhance the overall quality of aquatic feeds in an effective and sustainable way (Tao et al., 2023).

Yeast products, including yeast spent and yeast extract, are the by-products of the brewing and baking industries. Brewing spent yeast, as the second by-product of the brewing industry, has been reused as a functional ingredient to save fish meal in diets because of its higher contents in protein, polysaccharides, nucleotides, and other bioactive components. This is beneficial to both the brewing industry and aquaculture industry with regard to sustainability and environmental impact (Baiano, 2014). Many studies have reported that intact spent yeast was reused in aquafeed as an alternative protein ingredient to replace a fish meal (Tao et al., 2023). However, nutrients in spent yeast were not sufficiently utilized, owing to the thick cell wall of yeast, which blocks many bioactive components from being released for use (Baiano, 2014). Nevertheless, yeast extract is produced from spent yeast by disrupting the cell membrane with various methods (Demirgul et al., 2022). Indeed, yeast extract exhibits a notable release of polysaccharides, nucleotides, and other bioactive components compared to the intact spent yeast. Yeast extract is a water-soluble extract and is rich in peptides, free amino acids, nucleotides, ß-glucans, mannan oligosaccharides, B-complex vitamin, etc. (Tao et al., 2023). Peptides, amino acids, and vitamins are essential nutrients and play vital roles in the development of fish; nucleotides have been considered conditionally essential nutrients for fish under stress conditions or during rapid growth periods (Hossain et al., 2020); polysaccharides (e.g., ß-glucan and mannan oligosaccharides) are used as immunostimulants to better the intestinal health, immunity, and growth performance of fish and shrimp (Guzmán-Villanueva et al., 2013; Rajan et al., 2023). The beneficial bioactive components found in the yeast extract aid in enhancing the wellbeing of aquatic creatures and optimizing the effectiveness of aquafeed.

Earlier research studies have suggested that the addition of yeast extract to the diet resulted in an elevated specific growth rate and a reduced feed conversion ratio in some freshwater fish species, including snakehead fish (*Ophiocephalus argus*×*Channa maculata*) (Zhou et al., 2012), Gibel carp (*Carassius auratus gibelio*) (Chen et al., 2009), and Nile tilapia (*Oreochromis niloticus*) (Berto et al., 2016; Han et al., 2018). On the other hand, yeast extract did not show any notable effects on the growth of Chinese mitten crab (*Eriocheir sinensis*) and Pacific white shrimp (*Litopenaeus vannamei*), while the antioxidant capacities of shrimp and crab were improved by adding yeast extract in the feed (Zhang et al., 2019; Zheng et al., 2021). Moreover, yeast extract has been shown to be a superior alternative protein resource to intact spent yeast in shrimp (Zhao et al., 2017). Therefore, yeast extract is now more accurately defined as a functional additive that can enhance the growth performance, immunity, and antioxidant ability of aquatic animals (Podpora et al., 2015; Zheng et al., 2021). Nonetheless, few studies have been reported about the application of yeast extract in the feed of marine cultured fish.

The turbot is native to Europe and has been introduced to China for over 30 years due to its fast growth gate, good adaptability to intensive industrial aquaculture, and high market acceptance (Zhang et al., 2023). To date, the turbot has become an important mariculture flatfish in China, yielding more than 100,000 tonnes per year (Ministry of Agriculture and Rural Affairs of the People’s Republic of China, National Aquatic Technology Extension Station, China Society of Fisheries, 2023). Simultaneously, the culture of turbot in China is also facing the challenge of sustainable development because of the shortage of dietary protein sources and a high disease outbreak rate under intensive conditions. The effects of single components extracted from spent yeast, such as ß-glucans (Jiang et al., 2019; Gu et al., 2021), mannan oligosaccharides, and nucleotides (Fuchs et al., 2015; Bai et al., 2017), on the turbot have been assessed in several studies. Furthermore, the recent studies by Yang et al. (2020) and Wang et al. (2021) have illustrated the effectiveness of yeast cell wall extract in reducing toxins in turbot feed. Nevertheless, the influence of yeast extract, as a practical feed additive with several bioactive components, on the physiological status of turbot is still unknown. Hence, the objective of this investigation was to evaluate the effects of dietary yeast extract on growth, feed utilization, intestinal health, digestive ability, serum biochemical indexes, and antioxidant potential in turbot. The findings from this research will offer a valuable proof for the application of yeast extract in practical diets of turbot to improve the feed efficiency.

## 2 Materials and methods

### 2.1 Experimental diets

Yeast extract (YE) was obtained from Guangzhou Xintun Aquatech Co., Ltd. (Guangzhou, China); it contained moisture 57.30%, crude proteins 20.18%, small peptides 7.99%, ß-glucans 8.5%, mannan oligosaccharides 5.2%, nucleotides 3.2%, and amino nitrogen 1.1% (Table 1). Four isonitrogenous and isocaloric diets were formulated with graded levels of YE in 0 (YE0), 1.00% (YE1), 3.00% (YE3), and 5.00% (YE5). The formulation and proximate composition of experimental diets are listed in Table 2. To assess the apparent digestibility of nutrients, an inert marker consisting of 0.1% Y_2_O_3_ was incorporated into every diet. All feedstuffs were carefully weighed, mixed together, and ground before passing through a mesh sieve with a diameter of 178 µm. Next, the oil was added to the powder and thoroughly mixed together. The 2-mm soft pellets were produced using a pelletizer (EL-260, Weihai Youyi Factory, Shandon, China) and stored at −20°C until use.

**TABLE 1:** Composition of yeast extract used in this experiment (%, wet weight basis).

Component	Content (%)	Component	Content (%)
Moisture	57.30	ß-Glucan	8.50
Crude protein	20.18	Mannan oligosaccharide	5.20
Small peptide	7.99	Nucleotides	3.20
Amino nitrogen	1.10		

Yeast extract was purchased from Guangzhou Xintun Aquatech Co., Ltd. (Guangzhou, China).

**TABLE 2:** Ingredients and proximate composition of experimental diets of the turbot (%, dry matter basis).

*Ingredient*	YE0	YE1	YE3	YE5
Fish meal^a^	45.00	45.00	45.00	45.00
Meat and bone meal^a^	10.00	10.00	10.00	10.00
Squid meal powder^a^	4.00	4.00	4.00	4.00
Extruded soybean^a^	6.00	6.00	6.00	6.00
Corn gluten meal^a^	2.00	2.00	2.00	2.00
Cotton seed meal^a^	2.75	2.19	1.11	0
α-Starch^a^	14.00	14.00	14.00	14.00
Soybean oil^a^	2.50	2.50	2.50	2.50
Fish oil^a^	2.50	2.50	2.50	2.50
Calcium monophosphate^a^	4.50	4.50	4.50	4.50
Limestone powder^a^	0.50	0.50	0.50	0.50
Yeast extract^b^	0	1.00	3.00	5.00
Zeolite meal^a^	4.15	3.71	2.79	1.90
Mineral–vitamin premix^c^	2.00	2.00	2.00	2.00
Yttrium oxide (Y_2_O_3_)	0.10	0.10	0.10	0.10
*Proximate composition*				
Crude protein	45.73	45.80	45.02	45.03
Crude lipid	7.48	7.68	7.83	7.84
Crude ash	18.60	17.95	18.20	18.49
Gross energy/(MJ/kg)	18.12	18.45	18.19	18.56

^a^ These ingredients were provided from Hebei Haitai Tech. Ltd. (Shijiazhuang, China).

^b^ Yeast extract was purchased from Guangzhou Xintun Aquatech Co., Ltd. (Guangzhou, China).

^c^ Mineral–vitamin premix: according to Zhang et al. (2023).

### 2.2 Fish and feeding trial

Juvenile turbots were acquired from Minfeng Aquafarm (Tianjin, China) and adapted to experimental conditions for 14 d. During this time, they were fed with diet YE0. Subsequently, 2400 healthy turbots (average initial body weight of 4.2 ± 0.1 g) were randomly placed into 12 rectangular fiberglass tanks (200 cm × 100 cm × 100 cm), with 200 fish per tank. The tanks were provided with constant aeration. Before a formal meal, the feces and other waste in each tank were cleaned out. The fish were fed by hand with their specific diets twice a day (at 8:30 a.m. and 17:30 p.m.) to apparent satiation. In order to accurately measure the feed intake, the remaining feeds were siphoned out to collect, dry, and weigh 0.5 h post-feeding. Feces were collected from the third week, and the fresh feces were suctioned out after 1 h of feeding. Feces in one tank were pooled together and preserved at −20°C for the purpose of analyzing nutrient digestibility. The feeding trial was conducted under controlled environmental conditions, with the water temperature maintained between 15°C and 18.5°C, salinity levels ranging from 20‰ to 25‰, dissolved oxygen levels maintained above 6.0 mg/L, and ammonia–nitrogen levels kept below 0.5 mg/L. The duration of light was adjusted to 14 h, and the period of darkness was set to 10 h. The duration of the feeding trial was 8 weeks.

### 2.3 Sample collection

After an 8-week feeding trial, all fish were subjected to 24-h starvation and then were anesthetized using a solution of MS-222 (100 mg/L, Sigma-Aldrich, MO, United States). They were carefully weighed and counted. Afterward, four fish from each tank were randomly chosen and stored at −20°C in a refrigerator for the purpose of body composition analysis. Two fish per tank were selected to measure the body weight and then dissected on ice to obtain samples from the liver and viscera. The liver and viscera were weighed accurately to calculate the hepatosomatic index (HSI) and viscerosomatic index (VSI). Subsequently, the liver was stored at −20°C in a refrigerator for the analysis of antioxidant parameters. Moreover, two more fish in each tank were taken for blood samples using 1-mml syringes via the caudal vein. Serum was collected by centrifugation at 3000 *g* for 10 min and stored under −20°C for measuring physiological and biochemical parameters. The phlebotomized turbots were dissected to separate the anterior part of the gut for measuring the activities of digestive enzymes. Simultaneously, sections of the mid-intestine of approximately 1 cm were excised and placed in a solution of 4% paraformaldehyde for the purpose of histology examination. The intestinal segments (4-μm slides) were stained with hematoxylin and eosin (H&E) and used to observe the histological structures with a Zeiss microscope (Imager A1m, Oberkochen, GER).

### 2.4 Chemical analysis

All diets, fish bodies, and feces were ground into powder, and the powder was then examined for its proximate composition, including moisture, crude protein, crude lipid, crude ash, and gross energy, according to AOAC (2005) and Shi et al. (2022). The moisture content was determined using an oven at 105°C. Crude lipids were extracted using the Soxhlet method with petroleum ether. The Kjeldahl method was employed to measure the crude protein using a unit (4800 Kjeltec Analyzer, Hoganas, Sweden). The measurement of gross energy was conducted using a 6300 oxygen calorimeter (Parr instrument Company Moline, United States). The samples were incinerated in a muffle furnace at 550°C for a duration of 12 h to analyze the crude ash. An inductively coupled plasma source mass spectrophotometer (X Series 2 ICP-MS) (Thermo Fisher Scientific, United States) was used to measure the Y_2_O_3_ levels in both the diets and feces.

### 2.5 Physiological and biochemical index analysis

Serum levels of triglycerides (TGs), total cholesterol (TCHO), glucose (GLU), total protein (TP), blood urea nitrogen (BUN), alanine transaminase (ALT), aspartate transaminase (AST), and alkaline phosphatase (ALP) were analyzed using commercial kits (Leadman Biochemistry Company, Beijing, China) through an automatic biochemical analyzer (Mindray BS-180, Shenzhen, China). The activities of superoxide dismutase (SOD) and contents of malondialdehyde (MDA) in the liver were evaluated using the hydroxylamine method and thiobarbituric acid method, respectively, according to the guidelines of commercial kits (A001, A003; Nanjing Jiancheng Bioengineering Institute, Nanjing, China). The activities of intestinal trypsin, lipase, and amylase were measured using the colorimetric method with commercial reagent kits (A080, A054, C016, Jiangcheng Corp. Nanjing, China) using a microplate reader (BioTek Instruments, Inc., Winooski, VT, United States).

### 2.6 Statistical analysis

The data were present as means ± SE and analyzed with STATISTICA 10.0 software (StatSoft Inc., Tulsa, OK, United States). Confirmation of normality and variance homogeneity was conducted prior to performing statistical analysis. One-way ANOVA was conducted to assess the impact of yeast extract on all response variables, and in cases where a significant effect was detected in the ANOVA analysis, Duncan multiple comparisons were employed. The significance level was set at *p* < 0.05. In order to estimate the optimal level of yeast extract in the diets, a quadratic regression analysis based on the specific growth rate and graded levels of dietary yeast extract was employed.

## 3 Results

### 3.1 Growth performance

**FIGURE 1:**
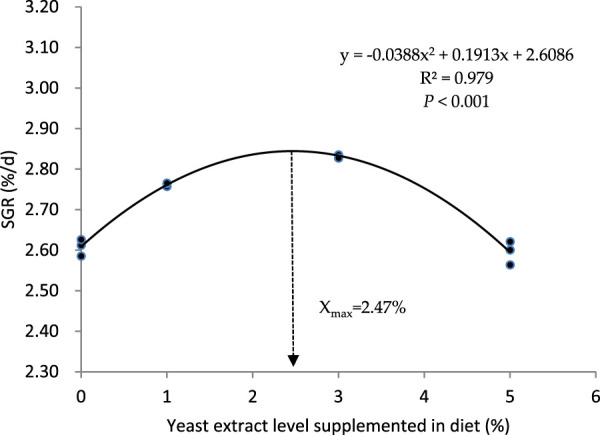
Quadratic regression relationship between the specific growth rate (SGR) of the turbot and yeast extract levels supplemented in experimental diets.

The growth, feed utilization, and body condition indexes of the turbot fed with graded levels of yeast extract are presented in Table 3. The survival rate of turbot in each group was 100%, and no dead fish were observed during the whole experimental period. Dietary supplementation with yeast extract significantly influenced the final body weight (FBW), weight gain rate (WGR), specific growth rate (SGR), feed conversion ratio (FCR), feeding rate (FR), and protein efficiency rate (PER) (*p* < 0.05). The values of FBW, WGR, and SGR in YE3 group were higher than those in other groups, and these parameters were distinctively higher in the YE1 group than those in the YE0 and YE5 groups (*p* < 0.05). The regression formula between SGR and dietary YE levels was determined as SGR =−0.0388YE^2^ + 0.1913 YE + 2.6086 (*R* ^2^ = 0.979, *p* < 0.05.), and the optimal supplemental level of YE in the diet was calculated as 2.47% based on this quadratic regression relationship (Figure 1). The YE3 group showed the lowest FCR among all groups, and the FCR of the turbot fed with diet YE1 was significantly lower than that of the turbot fed with the YE0 and YE5 diets (*p* < 0.05). FR in YE3 was higher than that in YE0 and YE5 groups, and group YE1 showed a lower FR than group YE5 (*p* < 0.05). Moreover, YE0 and YE5 displayed significantly inferior PER to YE1 and YE3 (*p* < 0.05). No significant differences in HSI and VSI parameters were observed among all treatments (*p* > 0.05). However, the values of CF in YE1 and YE3 treatments were significantly higher than those in YE1 and YE3 groups (*p* < 0.05).

**TABLE 3:** Effects of yeast extract supplemented in the diet on the growth performances of the turbot.

Parameter	YE0	YE1	YE3	YE5
IBW (g)	4.20 ± 0.00	4.24 ± 0.04	4.22 ± 0.04	4.25 ± 0.02
FBW (g)	18.09 ± 0.16^a^	19.93 ± 0.17^b^	20.64 ± 0.18^c^	18.15 ± 0.28^a^
WGR (%)	330.81 ± 2.87^a^	369.67 ± 0.69^b^	388.49 ± 0.70^c^	327.75 ± 4.00^a^
SGR (%/d)	2.61 ± 0.01^a^	2.76 ± 0.00^b^	2.83 ± 0.00^c^	2.60 ± 0.02^a^
FR (%/d)	1.85 ± 0.01^bc^	1.82 ± 0.01^ab^	1.79 ± 0.02^a^	1.88 ± 0.01^c^
FCR	0.83 ± 0.01^c^	0.79 ± 0.00^b^	0.76 ± 0.01^a^	0.84 ± 0.01^c^
PER (%)	257.89 ± 1.58^b^	272.19 ± 1.48^c^	279.66 ± 3.43^c^	250.30 ± 2.23^a^
CF	3.15 ± 0.65^a^	3.32 ± 0.77^b^	3.35 ± 0.73^b^	3.07 ± 0.3^a^
HSI (%)	1.52 ± 0.23	1.49 ± 0.06	1.46 ± 0.23	1.58 ± 0.10
VSI (%)	6.19 ± 0.65	6.23 ± 0.77	6.00 ± 0.73	6.49 ± 0.30
SR (%)	100	100	100	100

IBW, initial body weight; FBW, final body weight; weight gain rate (WGR, %) = [(final body weight–initial body weight)/initial body weight] × 100; specific growth rate (SGR, %/d) = [(Ln final body weight–Ln initial body weight)/days] × 100; feeding rate (FR, %/d) = 100 × feed intake/[days × (final body weight + initial body weight)/2]; feed conversion ratio (FCR) = [total dry feed intake (g)/wet weight gain (g)]; protein efficiency rate (PER, %) = [fresh body weight gain (g)/protein intake (g)] × 100; Fulton’s condition factor (CF) = 100 × [body weight (g)/body length (cm^3^)]; hepatosomatic index (HSI, %) = liver weight (g)/body weight (g) × 100; viscerosomatic index (VSI, %) = visceral weight (g)/body weight (g) × 100; survival rate (%) = final fish number/initial fish number. Data are presented as mean ± SE (*N* = 3). Means with different letters in a row are significantly different (*p* < 0.05).

### 3.2 Whole-body proximate composition

The moisture, crude lipid, and crude ash contents of whole body fish were not significantly different among all diets (*p* > 0.05) (Table 4). However, crude protein contents were significantly higher in the YE3 and YE5 groups than those in the YE0 and YE1 groups (*p* < 0.05).

**TABLE 4:** Effects of yeast extract supplemented in the diet on the whole body proximate composition of turbot.

Parameter (%)	YE0	YE1	YE3	YE5
Moisture	78.99 ± 0.22	78.53 ± 0.55	78.67 ± 0.68	78.70 ± 0.65
Crude protein	12.89 ± 0.27^a^	13.17 ± 0.26^a^	14.36 ± 0.28^b^	14.07 ± 0.37^b^
Crude lipid	2.96 ± 0.31	2.61 ± 0.25	2.46 ± 0.16	2.63 ± 0.07
Crude ash	3.50 ± 0.06	3.75 ± 0.19	3.47 ± 0.16	3.52 ± 0.21

Data are presented as mean ± SE (*N* = 3). Means with different letters are significantly different (*p* < 0.05).

### 3.3 Serum physiological and biochemical parameters

Dietary yeast extract supplementation has not markedly influenced the serum physiological and biochemical indexes (Table 5), and there were no significant differences observed in serum ALT, AST, TP, BUN, GLU, TG, and TCHO among all groups (*p* > 0.05).

**TABLE 5:** Effects of yeast extract supplemented in the diet on serum physiological and biochemical indexes of the turbot.

Parameter	YE0	YE1	YE3	YE5
ALT(U/L)	13.5 ± 2.81	14 ± 2.31	12.5 ± 3.91	13 ± 2.31
AST(U/L)	81.33 ± 10.7	96.50 ± 8.62	76.67 ± 15.53	85.50 ± 12.83
TP(g/L)	29.1 ± 1.11	29.4 ± 1.75	29.2 ± 2.75	29.8 ± 1.28
ALP(U/L)	8.17 ± 3.48	9.17 ± 4.98	8.50 ± 3.86	9.33 ± 3.35
BUN(mmol/L)	3.1 ± 0.45	3.3 ± 0.64	3.2 ± 0.59	3.6 ± 0.82
GLU(mmol/L)	2.04 ± 0.61	2.28 ± 0.38	1.93 ± 0.57	1.92 ± 0.26
TG(mmol/L)	1.55 ± 0.25	1.63 ± 0.18	1.35 ± 0.29	1.69 ± 0.33
TCHO(mmol/L)	1.92 ± 0.14	2.02 ± 0.15	1.82 ± 0.19	2.10 ± 0.19

ALT, alanine transaminase; AST, aspartate transaminase; TP, total protein; ALP, alkaline phosphatase; BUN, blood urea nitrogen; GLU, glucose; TG, triglyceride; TCHO, total cholesterol. Data are presented as mean ± SE (*N* = 6).

### 3.4 Digestion-related parameters

The digestion-related parameters of the turbot fed with four experimental diets are shown in Table 6. Yeast extract supplementations in diets led to a notable increase in the activities in contrast to the control group (YE0). Additionally, turbots in YE3 treatment exhibited higher levels of trypsin and amylase than those in YE0, YE1, and YE5 treatments (*p* < 0.05). Moreover, the apparent digestibility coefficient of dry matter (ADC_DM_) in the YE3 group was significantly higher than that in YE0 and YE5, and ADC_DM_ of YE5 was found to be the lowest among all treatments (*p* < 0.05). YE3 exhibits the greatest apparent digestibility coefficient of crude protein (ADC_CP_) among all treatments with a statistical difference, and the value of ADC_CP_ in the YE1 group was higher than that of YE5 significantly (*p* < 0.05).

**TABLE 6:** Effects of yeast extract supplemented in the diet on digestive enzymes activities and apparent digestibility coefficients of the turbot.

Parameter	YE0	YE1	YE3	YE5
*Digestive enzyme activities*				
Trypsin (U/mg prot)	486.10 ± 31.18^a^	508.08 ± 27.79^a^	613.93 ± 3.98^b^	546.32 ± 19.13^a^
Lipase (U/g prot)	21.12 ± 2.51^a^	36.13 ± 3.55^b^	43.36 ± 6.61^b^	32.84 ± 2.94^b^
Amylase (U/mg prot)	0.18 ± 0.02^a^	0.17 ± 0.01^a^	0.24 ± 0.02^b^	0.19 ± 0.01^a^
*Apparent digestibility coefficients*				
ADC_DM_ (%)	60.56 ± 0.80^b^	61.93 ± 0.08^bc^	63.01 ± 0.09^c^	58.96 ± 0.49^a^
ADC_CP_ (%)	84.50 ± 0.15^ab^	85.24 ± 0.58^b^	86.37 ± 0.20^c^	83.83 ± 0.44^a^

Data are presented as mean ± SE (N = 6 for enzyme activity; N = 3 for digestibility parameters). Apparent digestibility coefficient of dry matter (ADC_DM_, %) = (1 - Y_2_O_3_ content in diet/Y_2_O_3_ content in feces) × 100; apparent digestibility coefficient of crude protein (ADC_CP_, %) = 1–(crude protein content in feces/crude protein content in diet) × (Y_2_O_3_ content in diet/Y_2_O_3_ content in feces) × 100; means with different letters in a row are significantly different (*p* < 0.05).

### 3.5 Intestinal histology

**FIGURE 2:**
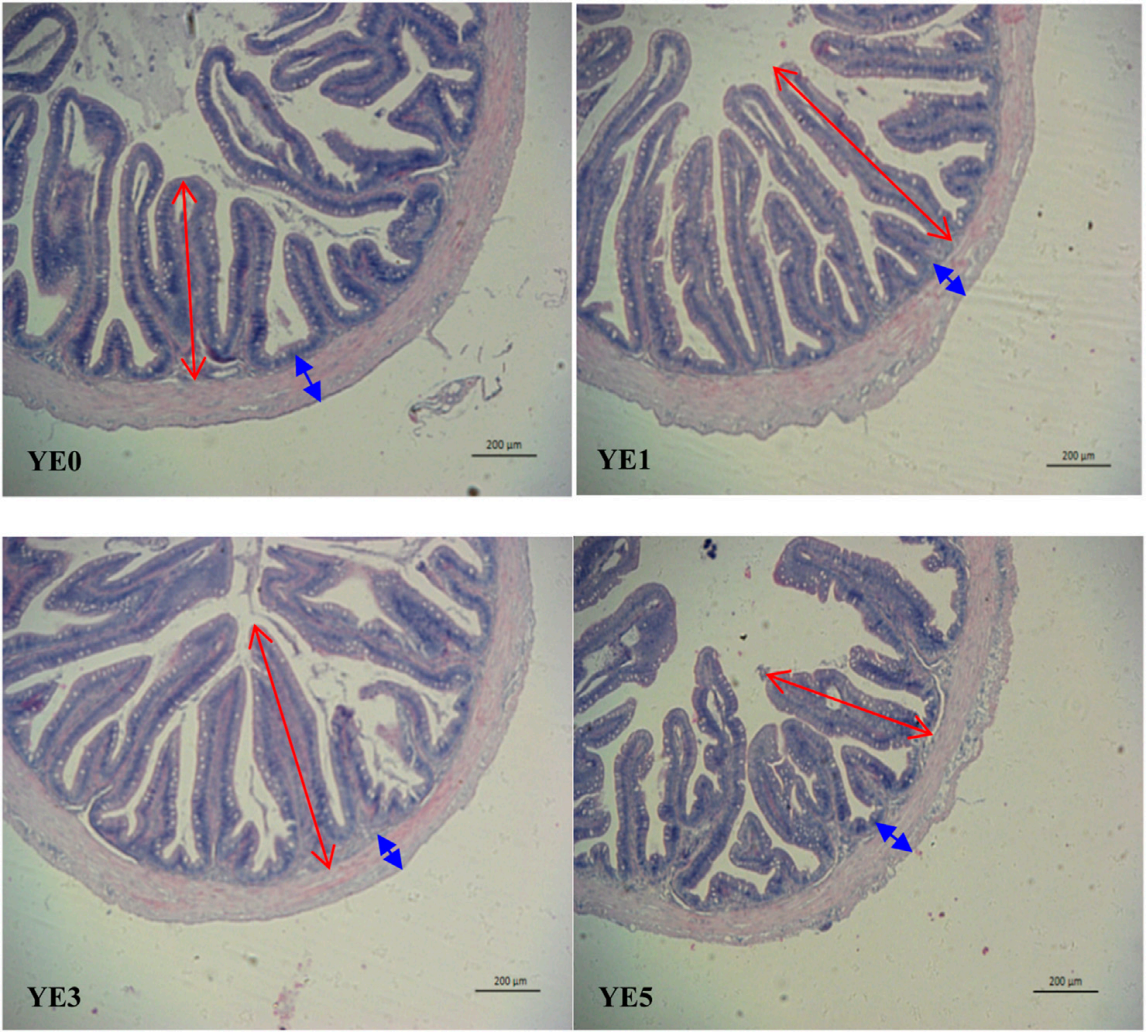
Mid-intestinal histology of the turbot fed four experimental diets with graded levels of yeast extract. The red arrow indicates the height of the intestinal fold, and the blue arrow indicates the thickness of the intestinal wall.

Figure 2; Table 7 display the mid-intestinal morphologies of the turbot fed with various experimental diets. Yeast extract had no different impact on the thickness of the intestinal wall (*p* > 0.05), while it significantly increased the height of mid-intestinal folds in YE1 and YE3 when compared with those in YE0 and YE5, and the fold height in YE3 was higher than that in YE1 treatment (*p* < 0.05).

**TABLE 7:** Effects of yeast extract supplemented in the diet on the intestinal histological structure of the turbot.

Parameter	YE0	YE1	YE3	YE5
Height of intestinal folds (μm)	484.82 ± 23.91^a^	546.13 ± 15.63^b^	616.86 ± 18.40^c^	439.09 ± 13.99^a^
Thickness of the intestinal wall (μm)	139.90 ± 5.04	122.62 ± 7.07	126.99 ± 6.65	118.78 ± 6.16

Data are presented as mean ± SE (N = 3). Means with different letters in a row are significantly different (*p* < 0.05).

### 3.6 Antioxidant-related parameters

**FIGURE 3:**
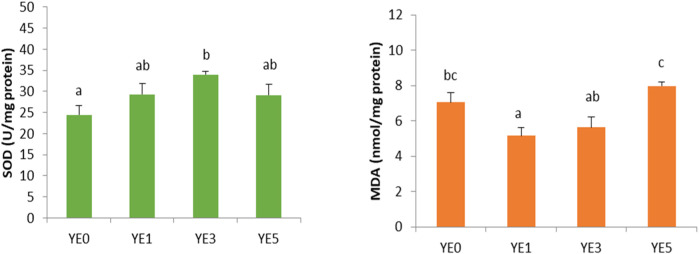
Effects of four experimental diets supplemented with graded levels of yeast extract on the anti-oxidant-related parameters in the liver of the turbot. SOD, superoxide dismutase; MDA, malondialdehyde. Data are presented as mean ± SE (*N* = 6). Means with different letters are significantly different (*p* < 0.05).

The SOD activities and MDA contents in the liver of turbot are exhibited in Figure 3. YE3 treatment displayed a significantly higher SOD activity than the YE0 group (*p* < 0.05). Additionally, YE1 exhibited a notable lower MDA content than YE0 and YE5, and YE3 had a remarkable lower MDA level than YE5 (*p* < 0.05).

## 4 Discussion

In this study, yeast extract has improved the growth and feed utilization of the turbot, which aligns with previous studies in Gibel carp, snakehead fish, and Nile tilapia, while differing from those observed in Pacific white shrimp and Chinese mitten crab (Chen et al., 2009; Zhou et al., 2012; Berto et al., 2016; Hassaan et al., 2018; Zhang et al., 2019; Zheng et al., 2021). The positive influence of yeast extract on the growth of turbot might be implicated with rich contents of bioactive components present in the yeast extract. The yeast extract utilized in this investigation was a product of crude extract paste from the brewing industry and comprised small peptides, ß-glucans, mannan oligosaccharides, nucleotides, and amino acids. Protein synthesis requires amino acids, which are also regarded as feed attractants in fish (Dabrowski et al., 2010), and the turbot was found to utilize small peptides more effectively than free amino acids (Wei et al., 2020). Nucleotides are intracellular compounds that play important roles in almost all biochemical processes. Although nucleotides can be produced from amino acids, they are insufficient during stress conditions and stages of rapid growth and development. Positive effects of nucleotides on growth and feed utilization have been demonstrated in the turbot (Li et al., 2018), beluga sturgeon (*Huso huso*) (Abtahi et al., 2013), rainbow trout (*Oncorhynchus mykiss*) (Tahmasebi-Kohyani et al., 2011), red drum (*Sciaenops ocellatus*) (Li et al., 2007), grouper (*Epinephelus malabaricus*) (Lin et al., 2009), and European sea bass (*Dicentrarchus labrax*) (Pelusio et al., 2023). The addition of nucleotides to the diet was discovered to enhance the expression of specific proteins involved in muscle metabolism and the structure in rainbow trout, along with insulin-like growth factor 1 in red sea bream (Keyvanshokooh and Tahmasebi-kohyani, 2012; Hossain et al., 2016). Moreover, polysaccharides are regarded as substances that enhance the growth and stimulate the immune system of fish and crustaceans (Mohan et al., 2019; Rajan et al., 2023). It is reported that dietary supplementation of mannan oligosaccharides has enhanced the growth and feed efficiency of turbots (Bai et al., 2017). Hence, these findings are helpful to understand the possible mechanisms of yeast extract in the aspect of contributing to good growth in turbots. However, an overabundance of nucleotides and polysaccharides in diets may result in a heightened immune response and hindered growth performance (Burrells et al., 2001; Hossain et al., 2020). Song et al. (2012) found that the growth of olive flounder (*Paralichthys olivaceus*) was hindered when fed a diet containing 1.0% IMP, in comparison to fish fed with 0.1%–0.2% IMP; dietary nucleotides at a high concentration (more than 1.5 g kg^-1^) inhibited the growth of red sea bream (Hossain et al., 2016). In the YE5 group of this study, the contents of nucleotides, ß-glucans, and mannan oligosaccharides in diet YE5 were calculated at 1.5 g kg^-1^, 4.25 g kg^-1^, and 2.6 g kg^-1^ in diets, respectively. They are at relatively higher levels according to previous results. Therefore, the decrease in turbot’s growth performance in YE5 treatment compared with the YE3 group may be due to the overdosing of these components in diet YE5. Turbot’s growth in this study showed a classic dose-dependent response to dietary yeast extract levels, and the appropriate dietary yeast extract content for improving the growth of turbot is 2.47% based on the quadratic regression relationship between SGR and dietary yeast extract levels.

As for the body compositions of turbot in this research, solely the amount of crude protein notably increased with the increasing levels of dietary yeast extract. This could be attributed to the yeast extract’s abundance of amino acid and nucleotide components. Nucleotides are served as the fundamental units for DNA to produce protein. Dietary supplementation of yeast extract would enhance protein biosynthesis by controlling the levels of nucleotides and amino acids in the cells (Hossain et al., 2020). On the other hand, appropriate supplementations of nucleotides and amino acids are helpful in improving the utilization of protein in the diet, and the increased PER observed in YE1 and YE3 groups of this study has shed light on this issue. Consequently, the nucleotide-rich yeast extract in this study promoted the protein synthesis of the turbot and enhanced its body protein content.

The beneficial impact of yeast products at appropriate levels on the intestinal health of fish and crustaceans was reported in many literature works; in general, 1%–2% yeast extract supplementation has increased the fold height of the anterior intestine in snakehead fish (Zhou et al., 2012), and the addition of additives extracted from yeast exhibited modulatory effects on intestinal morphology, particularly enhanced the intestinal mid-intestinal villi length and lamina propria width in gilthead sea bream (*Sparus aurata*) (Mallioris et al., 2022). Furthermore, the utilization of yeast and yeast extract led to a notable enhancement in the Shannon indexes of shrimp’s intestinal microbiota (Zheng et al., 2021). The substitution of fish meal with the yeast extract increased the nutrient digestibility of shrimp, as well as the hepatic trypsin activities (Zhao et al., 2017). Similar findings were found in our study, where 3% yeast extract enhanced the height of mid-intestinal folds, improved the digestibility of crude protein and dry matter, and increased the activities of trypsin, lipase, and amylase in the anterior intestine. Eventually, the intestinal health and feed utilization capacity of turbot were improved by dietary yeast extract addition. This may be attributed to the presence of mannan oligosaccharides and nucleotides in yeast extract. It was found that mannan oligosaccharides enhanced the growth and feed effectiveness while counteracting the negative impacts of soybean meal on intestinal wellbeing, which was achieved by boosting the performance of digestive enzymes and protecting against alterations in mucosal folds (Fuchs et al., 2015; Bai et al., 2017). Additionally, mannan oligosaccharides positively influenced the structure and microbial population of the gastrointestinal tract in rainbow trout and *Sparus aurata* (Dimitroglou et al., 2009; 2010). Regarding nucleotides, Hossain et al. have reviewed and summarized that nucleotides could enhance the intestinal health of fish by regulating the physiological and microbiological parameters in the gut (Hossain et al., 2020). It has also been found that nucleotides have upregulated the tight junction gene claudin 3 (*cldn3*) and induced the expression of interleukin 1β (i*l1b*) and interleukin 8 (*il8*) in the intestinal epithelial cell line of rainbow trout (Wang et al., 2019). Therefore, the addition of yeast extract also demonstrates a beneficial impact on the intestinal wellbeing of the turbot, similar to the majority of fish and shrimp. Dietary yeast extract improved the apparent digestibility of turbot by enhancing the histological structure of the intestines and increasing the secretion of various digestive enzymes.

The cells of the body can be harmed by highly reactive molecules known as superoxide anions, which are produced *in vivo* as a result of oxidative stress (Halliwell and Gutteridge, 1984). SOD is an important antioxidant enzyme that helps neutralize these harmful molecules by converting them into less-damaging hydrogen peroxide, which is helpful in preventing the accumulation of MDA and other detrimental by-products caused by oxidative stress in the body (Vlahogianni et al., 2007). In this study, the hepatic SOD activities in the YE3 group were significantly higher than those in control, and the MDA contents in YE1 and YE3 were relatively lower than those in the control group, which indicated that the yeast extract has increased the antioxidant capacity of turbots. The positive effects of yeast production on the antioxidant capacity were also observed in the studies of tilapia (Xu et al., 2015; Reda et al., 2018), shrimp (Xiong et al., 2018; Zheng et al., 2021), and crab (Zhang et al., 2019). Vieira et al. (2016) conducted a study to assess the antioxidant characteristics of brewer’s spent yeast extract using various assays, such as the DPPH (1,1-diphenyl-1-picrylhydrazyl) radical-scavenging capacity assay, ferricyanide-reducing power assay, and ferric-reducing antioxidant potential assay. They found that yeast extract displayed a favorable ability to counteract oxidation, despite the fact that the precise mechanism behind it remains unidentified. Furthermore, the healthy condition of fish was reflected by serum biochemical indexes. It is shown that no significant variations were observed in these parameters among all treatments in this study, which implies that yeast extract did not adversely impact the turbot’s wellbeing. Therefore, yeast extract has shown potential as a feed additive for the turbot, with promising results in terms of growth and digestibility promotion, and antioxidant properties.

## 5 Conclusion

In this study, the growth performance, feed conversion efficiency, body crude protein contents, apparent digestibility of nutrients, digestive enzyme activities, height of the intestinal fold, and the antioxidant capacity of juvenile turbots were significantly enhanced by the addition of yeast extract in the diet. The optimal growth and intestinal health of the turbot can be achieved by supplementing 2.47% yeast extract in the diet.

## Data Availability

The raw data supporting the conclusion of this article will be made available by the authors, without undue reservation.
